# TP53 R72P polymorphism modulates DNA methylation in hepatocellular carcinoma

**DOI:** 10.1186/s12943-015-0340-2

**Published:** 2015-04-02

**Authors:** Khadija Rebbani, Agnès Marchio, Sayeh Ezzikouri, Rajaa Afifi, Mostafa Kandil, Olfa Bahri, Henda Triki, Abdellah Essaid El Feydi, Anne Dejean, Soumaya Benjelloun, Pascal Pineau

**Affiliations:** Unité d’Organisation Nucléaire et Oncogenèse, INSERM U993, Institut Pasteur, 28 rue du Docteur Roux, F-75724 Paris, Cedex 15 France; Laboratoire des Hépatites Virales, Institut Pasteur du Maroc, 1 Place Louis Pasteur, 20360 Casablanca, Morocco; Service de Médecine C-Gastroentérologie, CHU Ibn-Sina, Rabat, Morocco; Equipe d’Anthropogénétique et de Biotechnologies, Faculté des Sciences Chouaib Doukkali, El Jadida, Morocco; Laboratoire de Virologie Clinique, Institut Pasteur de Tunis, Tunis, Tunisie

**Keywords:** TP53, DNA methylation, Hepatocellular Carcinoma, Polymorphism

## Abstract

**Background:**

Hepatocellular carcinoma (HCC) is characterized by widespread epidemiological and molecular heterogeneity. Previous work showed that in the western part of North Africa, a region of low incidence of HCC, mutations are scarce for this tumor type. As epigenetic changes are considered possible surrogates to mutations in human cancers, we decided, thus, to characterize DNA methylation in HCC from North-African patients.

**Methods:**

A set of 11 loci was investigated in a series of 45 tumor specimens using methylation-specific and combined-bisulfite restriction assay PCR. Results obtained on clinical samples were subsequently validated in liver cancer cell lines.

**Results:**

DNA methylation at tumor suppressor loci is significantly higher in samples displaying chromosome instability. More importantly, DNA methylation was significantly higher in Arg/Arg when compared to Pro/Pro genotype carriers at codon 72 rs1042522 of TP53 (65% vs 20% methylated loci, p = 0.0006), a polymorphism already known to affect somatic mutation rate in human carcinomas. *In vitro* experiments in cell lines indicated that enzymes controlling DNA methylation were differentially regulated by codon 72 Arg or Pro isoforms of p53. Furthermore, the Arg72-carrying version of p53 was shown to re-methylate DNA more rapidly than the pro-harboring isoform. Finally, Pro-carrying cell lines were shown to be significantly more resistant to decitabine treatment (two-fold, p = 0.005).

**Conclusions:**

Our data suggest that Arg72Pro polymorphism in a WT p53 context may act as a primary driver of epigenetic changes in HCC. It suggests, in addition, that rs1042522 genotype may predict sensitivity to epigenetic-targeted therapy. This model of liver tumorigenesis that associates low penetrance genetic predisposition to epigenetic changes emerges from a region of low HCC incidence and it may, therefore, apply essentially to population living in similar areas. Surveys on populations submitted to highly mutagenic conditions as perinatally-acquired chronic hepatitis B or aflatoxin B1 exposure remained to be conducted to validate our observations as a general model.

**Electronic supplementary material:**

The online version of this article (doi:10.1186/s12943-015-0340-2) contains supplementary material, which is available to authorized users.

## Background

Hepatocellular carcinoma (HCC), the most frequent primary liver tumor, is a malignancy affecting around 7 10^5^ patients each year, with highest incidences measured in Sub-Saharan Africa and Eastern Asia. HCC is now the fifth most common malignant tumor and the third common cause of cancer-related mortality worldwide [1]. The Western North-Africa (WNA: Morocco, Algeria and Tunisia), is considered as an area of intermediate endemicities for chronic viral hepatitis B and C, and displays much lower incidences of HCC than both European or African neighboring countries [2,3]. It is generally admitted that liver tumorigenesis is a consequence of accumulation of genetic and epigenetic alterations in key genes controlling proapoptotic or prosurvival signals. These changes occur generally in an impaired hepatic tissue undergoing a persistent viral infection and/or a cirrhosis, but it could be promoted also by an exposure to mutagens such as aflatoxin B1 [4]. Several classifications of HCC were established to differentiate tumors by focusing on histological characteristics, gene mutations in *TP53* and *Wnt* pathways and hypermethylation of tumor suppressor genes (TSG) [5,6]. In addition, HCC can be genomically characterized from the most instable tumors with frequent *TP53* and *AXIN1* mutations to stable tumors with *β-catenin* alterations [7]. Moreover, transcriptomics revealed an overexpression of imprinted and mitotic cell cycle genes within instable tumors at odds with stable ones that show increased levels of metabolism-controlling genes coupled to an underexpression of stress, immune response and cell adhesion coding genes [8,9]. Epigenetic alterations, and particularly DNA methylation, are also suspected to represent a class of decisive events in liver tumorigenesis. Indeed, diverse studies have been carried out on different cohorts of HCC patients affording insight about epigenetic changes controlling liver carcinogenesis [10]. Hypermethylation of a set of TSG such as *RASSF1, RIZ*, *CDK2NA* and *GSTP1* is commonly reported in HCC [11]. Such targeted silencing is generally accompanied by a global hypomethylation affecting repetitive elements covering the genome [12,13]. The connections between genetic and epigenetic features mentioned above are still poorly understood. Finally, numerous studies have been performed to explore the association of the genetic background of patients with an eventual individual susceptibility to HCC [14,15]. *TP53* presents, at codon 72, a functional single nucleotide polymorphism (SNP, R72P, rs1042522) that modulates the susceptibility to several cancers including HCC [16]. Notably, the presence of an arginine at codon 72 is known to be associated with higher rates of somatic *TP53* mutations in tumors [17].

Despite this apparent wealth of data, carcinogenesis in specific populations such as WNA inhabitants is still poorly understood. The WNA patients are, actually, characterized by the scarcity of alterations found in HCC [2]. This situation suggests that epigenetic changes may be the most significant changes in WNA patients. The aim of the study was, thus, to provide an appraisal of the epigenetic changes occurring in HCC from a WNA population. Methylation status at 10 individual loci as well as at repetitive elements (LINE-1) was assessed. Variations in DNA methylation levels were further confronted with genetic data including *β-catenin* and *TP53* mutations, chromosomal instability (CIN) and genotypes of selected SNPs. We found that, in HCC from WNA, somatic changes including methylation, mutations, or CIN were primarily conditioned by the genotype at codon 72 of *TP53*. The current report represents the first description of the existing correlation between *TP53 R72P* and epigenetic changes in tumors.

## Results

### Tumor specific patterns in HCC compared with the corresponding non-tumorous tissues

**Figure 1:**
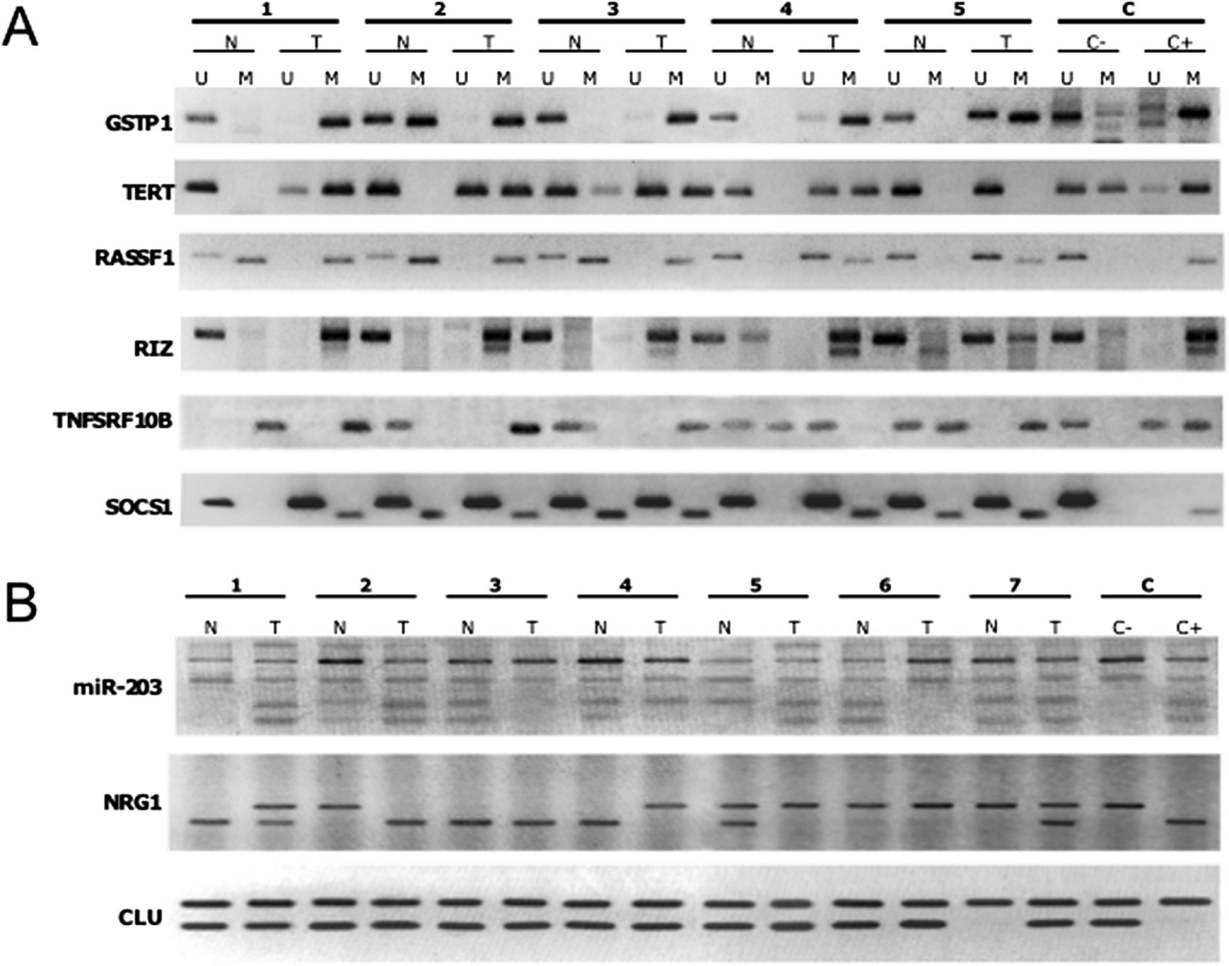
**Analysis of DNA methylation in WNA HCC by two PCR methods. (A)** Representative Methylation-Specific PCR for 6 of the 11loci on 5 different matching pairs of hepatocellular carcinomas and non-tumorous livers. U: PCR with primers for unmethylated DNA, M: PCR with primers detecting methylated DNA. **(B)** Representative examples of combined bisulfite restriction analysis (COBRA) performed on 7 pairs of hepatocellular carcinomas and corresponding non-tumorous livers on three different loci. The smaller bands beneath the larger ones represent digested (i.e. methylated for BstUI and unmethylated for HpHI restriction enzymes) alleles. N, noncancerous liver; T: livertumor, C: controls, C- : native DNA from WI38 fibroblasts in culture, C+: SssI CpG methylase-treated DNA from the same cells. Ratio of undigested/digested quantified with ImageJ 1.38 Instrument software integrated (Yang *et al.*, [75]).

Clinico-biological features of the HCC patients are summarized in Table 1. In this study, we examined methylation patterns of ten tumor-associated genes (*RASSF1A*, *GSTP1*, *RIZ1*, *SOCS1*, *TNFRSF10C*, *hTERT*, *NRG1*, *CLU*, *miR-203, miR-663*) and of the LINE-1 repetitive element in 45 HCC and 17 matching non-tumor livers (see Figure 1 and Additional file 1: Table S1). Genes were selected on the basis of the literature as aberrantly methylated in different human cancers including HCC [18-29].We define Methylation Index (MethIndex) as the number of methylated genes on the total number of informative loci analyzed for a given sample (LINE-1 was excluded from the calculation as it is constitutively methylated). m*iR-663* was found unmethylated in all samples.

**Table 1:** **Characteristics of the 45 patients analyzed**

**ClinicoBiological features**		**n**	**%**
Gender	Women	12	26.7
	Men	33	73.3
Age	mean ± SD	58.6 ± 11.6	
	median	60	
	range	28-79	
Risk factors	anti-HCV	26	58
	HBs Ag	10	22
	anti-HBc	20	44
Cirrhotic liver		38	84
Tumor diameter	mean ± SD	5.1 ± 3.6	
	median	4	
	range	1-13	
Point mutations	TP53	9	20
	β-catenin	4	9
	AXIN1	0	0
Chromosome instability	mean ± SD	29 ± 24	
	median	20	
	range	0-80	

Chromosome instability is expressed as the fraction of chromosome arms lost in a given samples (chromosomes 1p, 4q, 6q, 8p, 9p, 17p were analyzed).

HCV, hepatitis C virus; HBs Ag, surface antigen of hepatitis B virus; HBc, hepatitis B virus capsid/core.

**Figure 2:**
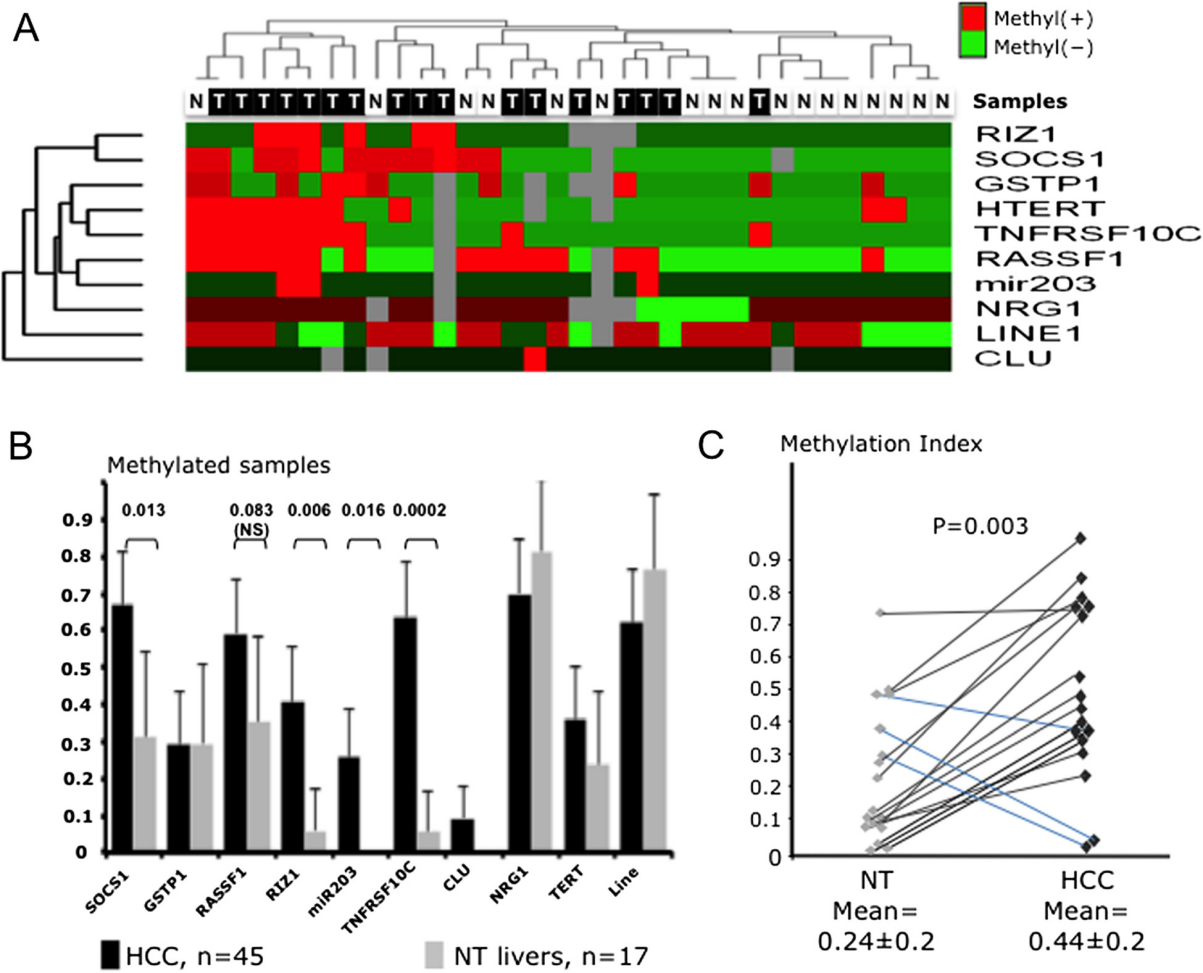
**Distinct DNA methylation levels in HCC and non-tumor livers. (A)** Hierarchical unsupervisedclustering and corresponding heatmap of the methylation status in 17 WNA HCCs and the 17 matching non tumorous livers. Clustering was obtained using a 1-rank correlation distance calculation and an average linkage on DChip software. T symbolyzes the tumor samples whereas N corresponds to the non-tumor liver counterparts. **(B)** Comparison of the methylation rates as detected at the different loci in the 45 HCC and the 17 Non-tumor livers. **(C)** Methylation Index in HCC and matching non-tumor liver DNA from the same patient. Lines link the dots corresponding to T and NT specimens of the same patient.

First, we intended to check whether there was a significant increase of DNA methylation in tumors when compared with non-tumorous DNA extracted from the liver tissue of patients with HCC. The methylation profiles of 17 HCCs and their matching non-tumorous liver (NTL) tissues were visualized on a heatmap produced by unsupervised hierarchical clustering approach. Major differences between tumors and non-tumorous DNA enabled clustering analysis to discriminate between sample types (see Figure 2A). Significant differences or trends between HCC and NTL were detectable at four loci (*SOCS1, RIZ1, TNFRSF10C*, *miR-203*) out of the 11 examined (Figure 2B). *RASSF1* locus comparison displayed only an infra-significant P value (P = 0.083). The overall difference between malignant and non-malignant tissues was highly significant (MethIndex was mean ± SD = 44 ± 20% in HCC versus 24 ± 20% in NT, p = 0.003, Figure 2C).

### Patterns of DNA methylation in HCC from WNA

**Figure 3:**
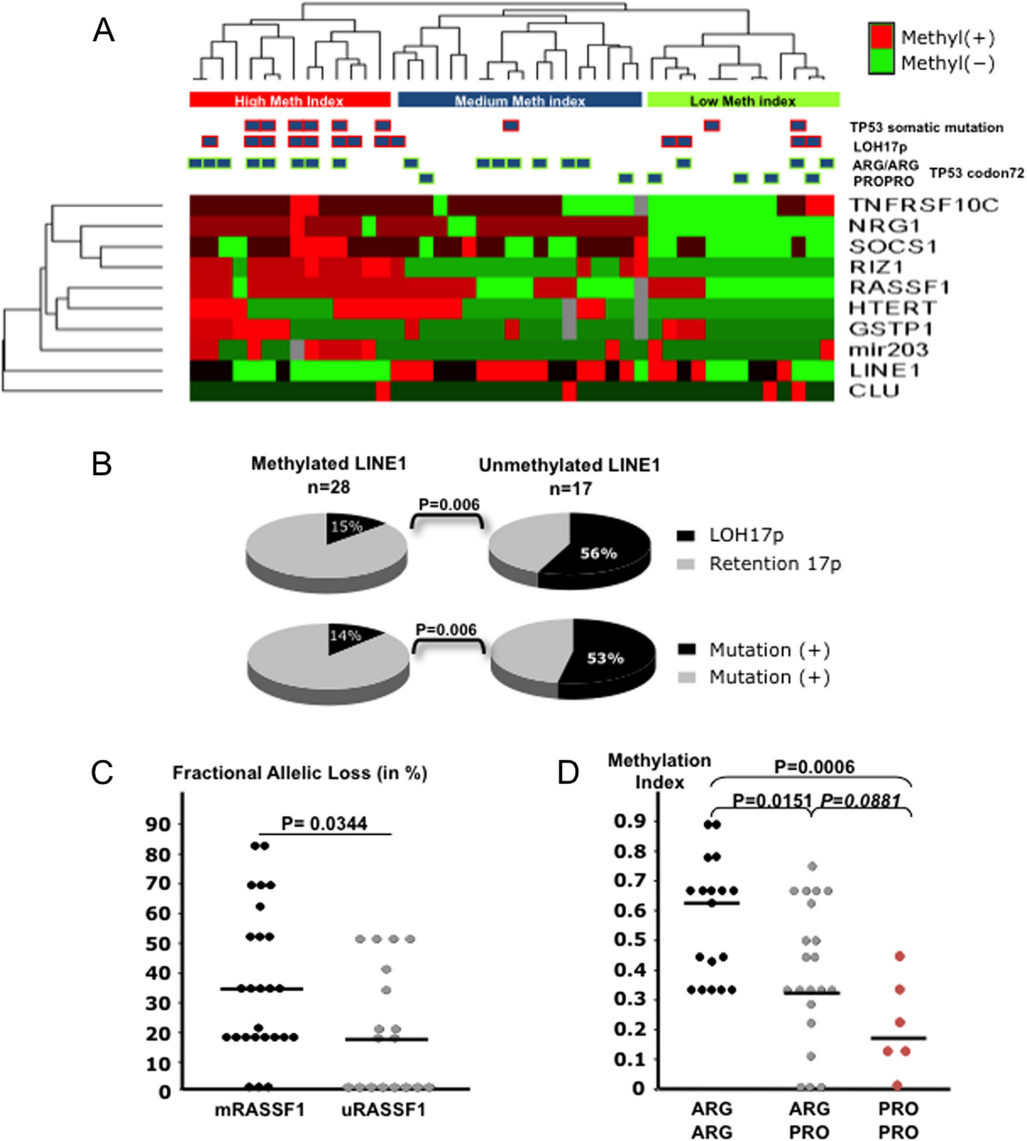
***TP53*** **ARG72PRO modulates DNA methylation in WNA HCC. (A)** Hierarchical clustering and corresponding heatmap of the methylation status at 10 loci in 45 WNA HCCs. Clustering was obtained using a 1-rank correlation distance calculation and an average linkage on DChip software. Squares indicate the presence of a somatic change or a pathogenic variant in the corresponding samples. *TP53* somatic mutation (p = 0.017), LOH at chromosome 17p (p = 0.0094) and chromosome instability (p = 0.067) are associated with the high methylation index cluster whereas Pro/Pro at codon 72 of *TP53* is significantly associated (p = 0.048) with a medium or low methylation index. **(B)** In WNA HCC, methylation of LINE elements correlates significantly with the presence of other somatic changes as point mutations (in *TP53*, *CTNNB1* and *AXIN1*) or allelic loss at chromosome 17p where is mapping *TP53*. **(C)** Distribution of the Fractional Allelic Loss (FAL) according to the methylation status of *RASSF1*. m*RASSF1*, methylated or hemimethylated locus; u*RASSF1*, unmethylated locus. FAL was assessed on 6 chromosomes (1p, 4q, 6q, 8p, 9p, and 17p). LOH, loss of heterozygosity. **(D)** Distribution of the methylation index according to the genotype at codon 72 of p53. Methylation index is calculated as the methylated fraction at 9 loci (*SOCS1*, *RASSF1*, *RIZ1*, *GSTP1*, *TNFRSF10C*, miR203, *NRG1*, *CLU* and *TERT*).

We then wondered whether the methylation pattern could distinguish different subsets of tumors in correlation with previously analyzed somatic mutations, loss of heterozygosity (LOH) or SNPs [2,30]. Unsupervised hierarchical clustering distinguished three categories of HCC patients: those with low MethIndex (mean ± SD = 19 ± 18%), a second group with medium MethIndex (42 ± 12%) and a third subset with high (69 ± 11%) MethIndex (Figure 3A). *TP53* somatic mutations and LOH at chromosome 17p (*TP53* maps in 17p13.1) were significantly associated with the high methylation index cluster (p = 0.022 and p = 0.014). Single-locus matrix of correlation revealed frequent significant associations between methylation occurring at *RIZ1* or *RASSF1* promoters and presence of methylation at other loci (Additional file 1: Table S2). Furthermore, increased methylation of tumor suppressor genes or decreased methylation of LINE-1 repetitive element, were associated with higher rates of somatic changes in tumor samples. There was a strong correlation between LINE-1 demethylation and the loss of chromosome 17p or the presence of somatic mutations in the tumor (either at *TP53* or *CTNNB1*, p = 0.006, see Figure 3B). We next decided to explore whether DNA methylation at individual loci could be correlated with a more general form of chromosome instability. We calculated in a previous work on the same samples the fractional allelic loss (FAL) for common deletion targets in HCC (1p, 4q, 6q, 8p, 9p and 17p) [2]. FAL is the number of chromosome arms showing LOH/number of informative chromosome in a given sample [31,32]. *RASSF1* methylated status was significantly associated with higher FAL values (p = 0.034, Figure 3C) as well as isolated 17p loss (Additional file 1: Table S3). Methylation of *RIZ1* and *miR-203* was positively correlated with somatic mutations or losses at chromosome 17p and 4q (see Additional file 1: Table S3). Finally, we noticed a strong influence of the SNP rs1042522 on the methylation of WNA HCCs. The proline variant at codon 72 of *TP53* gene was significantly associated with the low methylation cluster (p = 0.04, Figure 3A), and specifically with an almost complete absence of methylation on *RIZ1*, *TNFRSF10C, NRG1, hTERT* and *miR-203*. When the MethIndex was stratified according to *TP53* codon 72 genotypes, significant differences were even more apparent (see Figure 3D). A very low MethIndex was associated with Pro/Pro genotype (mean ± SD = 20.8 ± 14%) whereas methylation was significantly higher in tumors of individuals homozygous for arginine (57.1 ± 19%, P = 0.0006). Tumors from heterozygous Arg/Pro were occupying an intermediate position for MethIndex (39.1 ± 23%) suggesting the existence of an allele dosage effect of *TP53* codon 72 polymorphism on the DNA methylation in HCC from WNA patients. In addition, MethIndex distributions for Arg/Arg and Arg/Pro carriers were displaying a bimodal distribution (see Figure 3D). Methylation of *SOCS1* was appearing as particularly correlated with *TP53* codon 72 genotype (see Additional file 1: Table S3).

### DNA methylation in peripheral blood mononuclear cells (PBMCs) of HCC patients and controls

Predicting the propensity of individuals to undergo a strong DNA methylation in their tumors may prove to be useful in a perspective of future treatment by epigenetic-targeting drugs. To determine whether it is possible to find a correlation between the aberrant methylation process ongoing in tumors and the DNA methylation in circulating lymphocytes, we analyzed the DNA extracted from peripheral lymphocytes of 25 patients with HCC and 35 patients with chronic hepatitis B or C but without HCC. All patients were from WNA. No significant differences were detected between patients with or without primary liver cancer (see Additional file 2: Figure S1A). DNA Methylation was very low in PBMCs of HCC patients as well as in patients infected by hepatitis viruses (Additional file 2: Figure S1B). In contrast, LINE-1 methylation remained high in both subsets of samples. Genetic targets usually used to assess the methylation process in tumors did not appear to be sensitive markers reflecting the tumor DNA methylation level when they were analyzed ectopically as in PBMC (Additional file 2: Figure S1C).

### DNMT and DNA demethylases expression levels in liver cancer cell lines according to TP53 codon72 genotype

**Figure 4:**
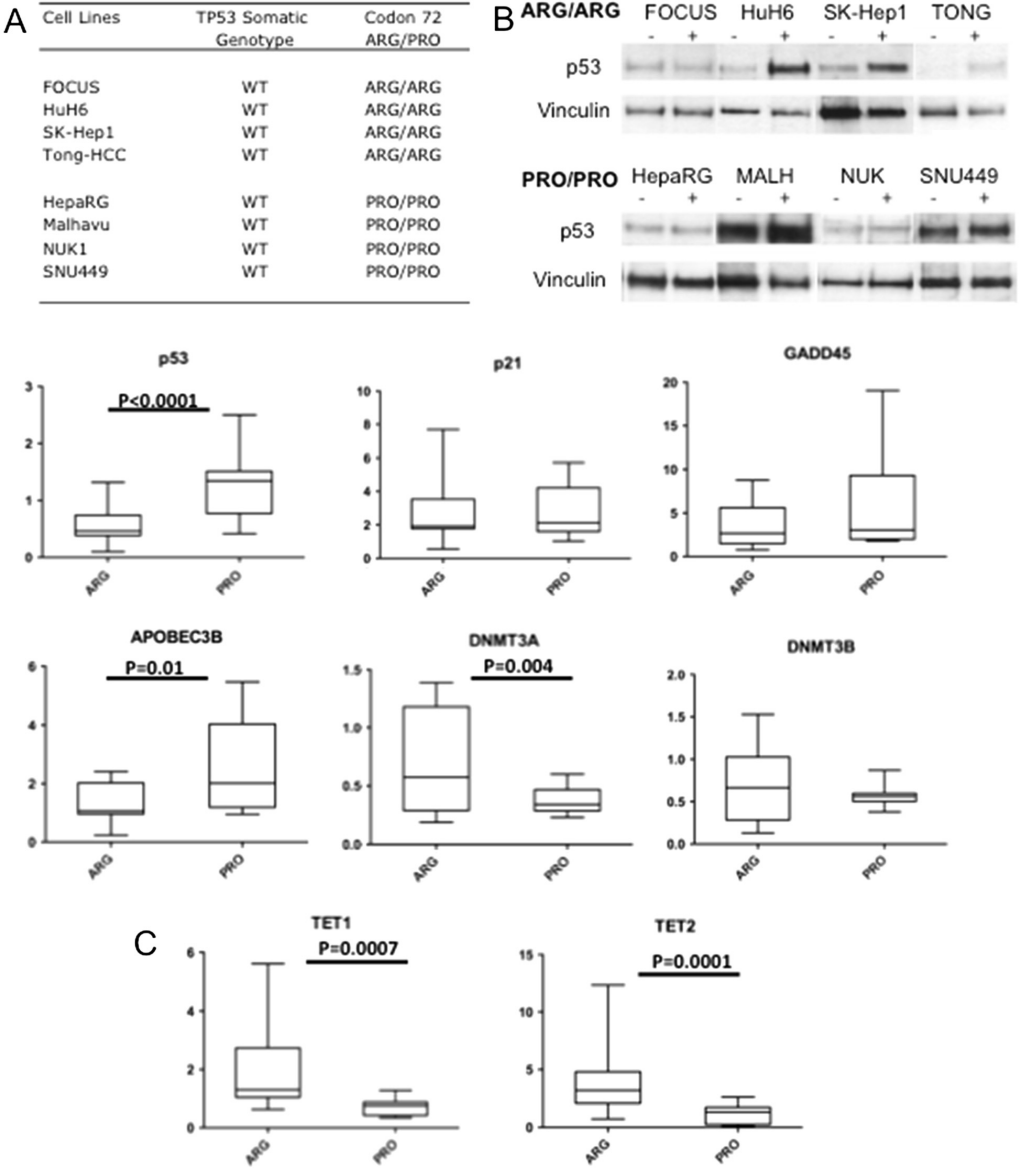
**Impact of** ***TP53*** **ARG72PRO on the expression of genes involved in DNA methylation metabolism. (A)** Panel of liver cancer cell lines wild-type for *TP53* used for *in vitro* experiments. **(B)** Western-blot analysis of p53 expression (baseline and doxorubicin-induced) in the panel of cell lines. +: indicates doxorubicin treatment, 0.2 μM/72 h. **(C)** qRT-PCR analysis of genes involved in DNA methylation metabolism. All cells were treated 48 h by 0.5 μM doxorubicin. P values are obtained by ANOVA analysis.

We decided subsequently to explore *in vitro* whether p53 codon 72 Arg or Pro-encoding isoforms could be endowed of a differential transcriptional activity, explaining the rate of DNA methylation observed *in vivo*. For this purpose, we analyzed eight different *TP53* wild-type HCC cell lines differing at codon 72 (Figure 4A). All these cell lines were expressing p53 protein either at baseline or upon induction by doxorubicin (0.2 μM/24 h, see western-blot on Figure 4B).

*TP53* encodes a transcription activator/repressor known to modulate directly or indirectly expression levels of many genes [33]. As the codon 72 Arg72Pro polymorphism is located in the transactivation domain of the protein, we wondered whether this genetic variation might impact the capacity of p53 to modulate expression of those genes most directly involved in DNA methylation i.e. DNA methyltransferases and demethylases [34,35]. Using qPCR, we compared the expression of sixteen genes encoding for factors modulating DNA methylation levels positively or negatively. Gene expression was measured after 0.5 μM doxorubicin, or 2.5 μM decitabine or vehicle treatment for 48 h. Baseline or post-decitabine expression of the 16 genes was not different according to *TP53* codon 72 genotypes (data not shown). Following doxorubicin treatment, we did not observe difference for two *bona fide* p53-responsive genes (p21-*CDKN1A*, *GADD45*). By contrast, *DNMT3A, TET1 and TET2* expression levels were significantly higher in Arg/Arg cell lines whereas in a Pro/Pro background *APOBEC3B* expression was increased (see Figure 4C). Importantly, *TP53* expression was significantly higher in Pro/Pro cells. These data suggest that, in case of genotoxic stress, Arg-bearing isoforms of p53 are more efficient inducers of enzyme controlling DNA methylation than Pro-encoding isoforms.

### Impact of TP53 codon 72 Arg/Pro on DNA methylation levels

We next explored whether it is possible to mimic in cell lines what we observed on DNA from North-African HCC patients *i.e.* an increased DNA methylation in presence of Arg/Arg genotype. At variance with commonly performed gene inactivation experiments, differences of activity between two natural variants of a fully functional protein were predictably rather mild [36]. Furthermore, the DNA methylation status is intrinsic to each cell line and may differ widely at baseline. We thus decided to analyze DNA methylation and re-methylation dynamics following treatment with drugs modifying DNA methylation levels. Decitabine alone, doxorubicin alone or both drugs were sequentially used. Doxorubicin, as an intercalating agent, is known to induce DNA breaks but early reports have shown as well its capacity to induce DNA methylation [32]. To analyze methylation variations, we used a methylation-specific restriction enzyme (MSRE)-coupled qPCR method [37,38]. A set of seven CpG island-containing promoters (*BIRC5*, *CDC25C*, *CLU*, *GSTP1*, *NRG1*, *RASSF1*, *SOCS1*) was investigated on the panel of *TP53* wild-type codon 72 Arg or Pro HCC cell lines (Additional file 3: Figure S2).

**Figure 5:**
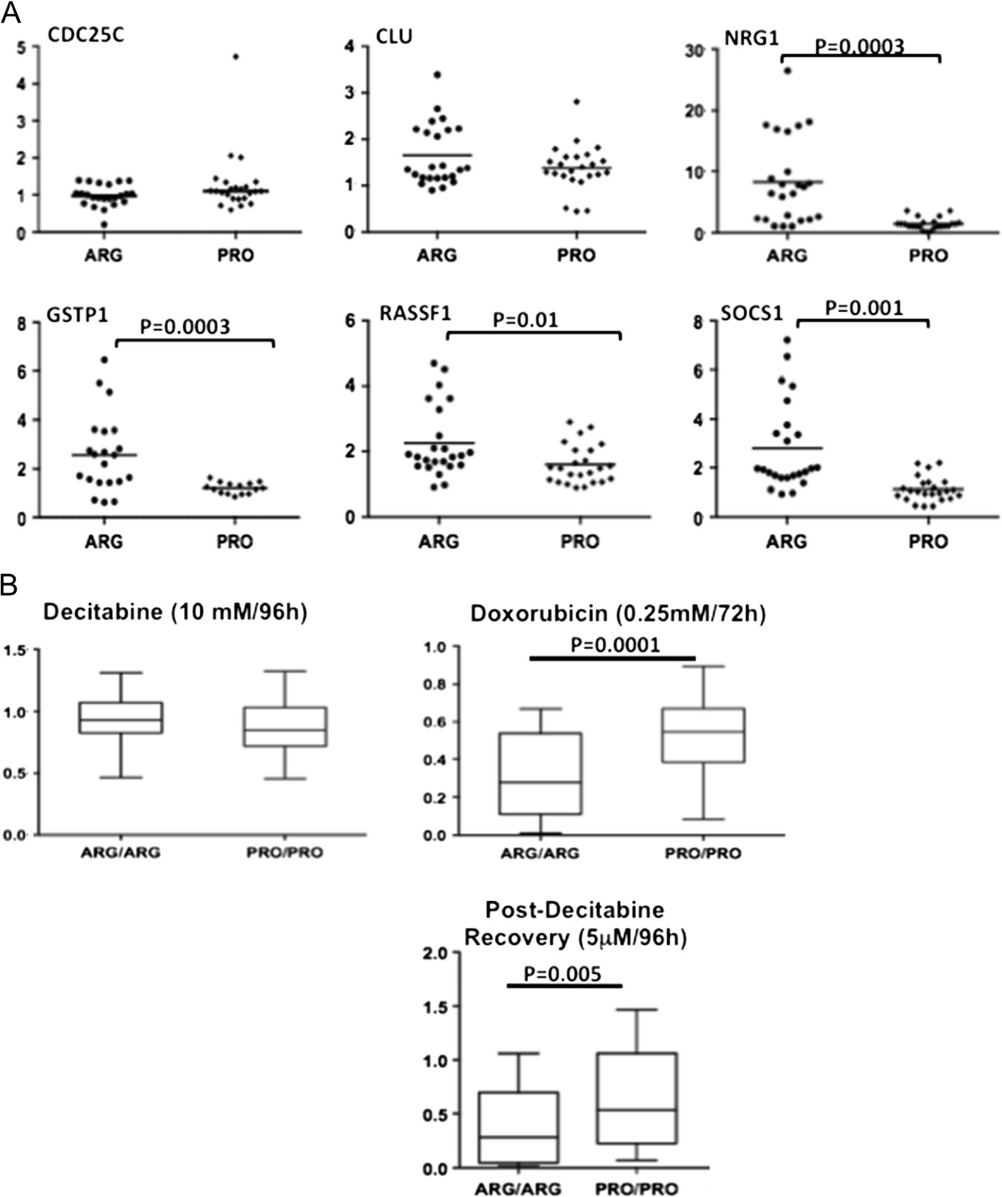
**Increased DNA methylation and increased sensitivity to DNA demethylation of ARG72 carrying HCC cell lines. (A)** MSRE-coupled qPCR analysis following sequential treatment of the cells by decitabine (5 μM/72 h) and doxorubicin (0.25 μM/24 h). ARG/ARG cell lines are often more resistant to methylation-sensitive DNA restriction than PRO/PRO cells that usually yield weaker signal in qPCR. The figure represents the outcome of two independent experiments in triplicate. Fold is relative to decitabine-treated only DNA. **(B)** Cell viability analysis of Cell lines as measured by MTT assay in different treatment conditions.

The impact of the treatment was dependent on the compound and on the promoter analyzed. No difference in DNA methylation level was observed after doxorubicin or decitabine treatment alone (data not shown). However, when decitabine (5 μM/72 h) and doxorubicin (0.25 μM/24 h) were used sequentially in a demethylation-remethylation procedure, a frequently higher rate of DNA methylation was observed (see Figure 5A) in presence of codon 72 Arg forms of p53, indicating a more active kinetic of DNA methylation. The phenomenon was particularly pronounced for genes displaying differential methylation *in vivo* (*GSTP1*, *NRG1*, *RASSF1*, and *SOCS1*). It was not observed on promoters of genes without differential methylation (*CLU*) or controlling cell cycle (data of *CDC25C, BIRC5* are not shown).

### TP53 codon 72 polymorphism affects sensitivity to treatment

Given the differential gene expression or DNA methylation capacity of p53 variants, the issue of a differential sensitivity to epigenetic-targeted treatment was asked. The eight *TP53* WT cell lines were, thus, treated either with decitabine (10 μM/96 h) or doxorubicin (0.25 μM/72 h) and cell viability measured by MTT assay. No difference was observed with decitabine whereas homozygous Arg cells were significantly more sensitive to doxorubicin treatment (Figure 5B). Previous experiments have shown that differential *in vitro* effect of codon 72 polymorphism was essentially a kinetic-dependent phenomenon rather than relying on a difference in nature. We thus decided to measure the effect of decitabine at different time point after removal of the active compound. Cells were treated by 5 μM decitabine during 96 h and cell culture medium was then replaced by decitabine-free medium. Cell viability was then measured at 24 h, 48 h, 72 h, 96 h, 7 days and 10 days. No difference was observed after 24 h or 7 and 10 days of recovery (data not shown). However, significant differences of cell viability were observed 48 h (p = 0.05, not shown), 72 h (p = 0.01, not shown), and 96 h (p = 0.005, see Figure 5B) post-decitabine removal. In these settings, Arg/Arg HCC cells were significantly less viable than Pro/Pro. These results suggest, therefore, a more efficient death-inducing activity of decitabine in an Arg-bearing p53 background.

## Discussion

HCC epidemiology is known to follow ethno-geographic variations of incidence. Intriguingly, despite intermediate endemicities for chronic viral hepatitis B and C, low incidences of HCC are registered in the WNA populations (1.2-2.3 cases/10^5^ habitants for males, age standardized ratio, http://globocan.iarc.fr/) [39,40]. Remarkably, in WNA, HCC is characterized by a relative paucity of mutations at common genetic targets (*TP53*, *CTNNB1*, *AXIN1*), making of liver tumorigenesis in WNA a rather mysterious process (2). Nevertheless, and despite an apparently mild tumor process, HCC prognosis in WNA is as bad as anywhere else [41,42].

To date, no survey characterizing epigenetic features of HCC in WNA patients has been published. Our results were in accordance with those published by Hernandez-Vargas *et al*. underlining the importance of aberrant methylation that differentially affects *RASSF1*, *RIZ1* and *SOCS1* in tumors and non-tumorous tissues [43]. A rather high level of methylation was observed in four NT liver DNA (≥4 loci methyl(+), see Figure 2A). We could not find any annotations common to the four patients. However, we observed that their non-tumor liver tissues tended to be more frequently non cirrhotic (3/4 vs 5/41, p = 0 .014) and the FAL in tumors slightly higher (38 vs 28%, ns) than in other samples of the series. These observations might indicate that a stronger carcinogenic process conferring a preneoplastic status to NT liver is at work in these patients. Alternatively, it might be the consequence of a field cancerization process in absence of full-fledged cirrhosis [44-46]. Besides, Nishida *et al.* have shown that DNA methylation levels are correlated with chromosomal instability, *TP53* and *β-catenin* mutations [47]. Our data broadly corroborated this model, though without integrating *β-catenin*, as this alteration is very infrequent in WNA HCC. We hereby presented data refining and strengthening this model by showing a link between polymorphism at codon 72 of *TP53*, somatic mutations, chromosomal instability and DNA methylation status in HCC from WNA patients. The preferential association of codon 72 Arg with somatic mutation of *TP53* has been thoroughly reviewed by Soussi and Wiman [17]. Likewise, the well-known association of chromosomal instability and *TP53* mutation will preferentially occur in an Arg/Arg context [48]. Finally, it has been shown that DNA methylation is significantly reduced in Li-Fraumeni cell lines [49]. However, to our best knowledge, a correlation between aberrant methylation and germline background of patients for *TP53* was never reported.

The *TP53* R72P polymorphism is known to be functionally relevant. The Arg variant was shown to be associated with a better apoptotic activity compared to Pro allele [50]. Furthermore, predisposition studies showed that the presence of the Pro allele is commonly associated with a higher risk of cancer including HCC [30,51] and of defects in embryonic implantation [52]. Our data indicate that codon 72 polymorphism conditions, apparently, genomic and epigenetic alterations encountered in the tumor tissue. In the current series of HCC from WNA patients, the Pro variant is associated with paucimutated and mildly methylated tumors. On the contrary, the presence of Arg allele requires the presence of additional somatic mutations and frequent DNA hypermethylation. It is well known that aging process is accompanied by an increase of genomic methylation that progressively decreases the expression of multiple genes [53]. Moreover, as shown in mouse models, an excess of p53 activity is characterized by an aging-like syndrome [54]. Finally, it has been shown in humans that *TP53* codon72 Pro carriers tend to live longer than Arg carriers [55]. A link between rs1042522 and constitutive DNA methylation was, however, never evoked so far. Our results indicated consistently that *TP53* codon72 Arg/Pro polymorphism may, in the North-African ethno-environmental settings, influence somatic evolution through the modulation of DNA methylation levels. The fact that we were able to reproduce this situation *in vitro* reinforces this hypothesis. We did not detect baseline differences of DNA methylation between cell lines but an increased DNA methylation was often detectable in Arg/Arg cells after decitabine-induced demethylation and subsequent doxorubicin-induced DNA re-methylation. Interestingly, three of the genes showing differential methylation between Arg and Pro variants (*GSTP1*, *RASSF1*, and *SOCS1*) are known to be p53 targets [56-58]. This situation suggests that p53 may, depending on circumstances activates or methylate its target genes. The system we developed *in vitro* is relatively removed from physiological conditions or from a slow and long lasting process as tumorigenesis but it suggests that p53 controls DNA-methylation plasticity in condition of stress. Genome wide DNA methylation studies on normal and pathological conditions are now warranted to confirm our data. Another intriguing feature of DNA methylation in Arg/Arg and Arg/Pro carriers was its bimodal distribution. We were not able to correlate this feature with any of the clinic-biological annotations at our disposal. It is, thus, tempting to hypothesize that p53 impact on DNA methylation could be modulated by another genetic trait. It is, indeed, well known that DNA methylation levels in humans are strongly influenced by polymorphisms affecting genes controlling one-carbon metabolism [59,60]. Moreover other polymorphic effectors of DNA methylation, to be found in the oxidative stress pathway or among partners acting directly in DNA methylation (*eg* DNMTs), can be suspected to influence methylation levels [61-63].

Being aware that we do not provide a mechanistic explanation to our observations, some conspicuous differential expression affecting genes involved in DNA methylation metabolism, particularly those of *DNMT3A* (methylating) or *APOBEC3B* (demethylating), however, are in keeping with the current model of DNA-methylation metabolism [64]. It is well known that p53 entertains tight connections with the different cellular DNA-methyltransferases. Indeed, p53 and Dnmt3A are direct interactors as shown by co-immunoprecipitation experiments [65,66]. In addition, and in absence of mutagenic stress, p53 is known to directly inhibit *DNMT1* expression by trapping Sp1 and repressive chromatin modifiers on the promoter of the gene [67,68]. The links between p53 and Dnmt1 are, however, far more complex. Both proteins have been shown to physically interact on various p53-responsive promoters (*eg* survivin/*BIRC5*) leading to their inactivation through DNA methylation as well as other chromatin modifications [38,69]. These data and the well-described differential activities of p53 Arg/Pro isoforms in other epigenetic-sensitive phenomena such as cancer or ageing, make plausible a differential activity of rs1042522 on DNA methylation levels.

Finally, we showed a mild but significant increased ability to recover from decitabine treatment in Pro-carrying cells than in Arg carriers. This is in line with our hypothesis according to which tumorigenesis is less dependent on DNA methylation in Pro than in Arg carriers. In addition, and despite hitherto disappointing results in solid tumors, our data suggested that decitabine use in a codon 72 Arg/Arg wild-type TP53 context might significantly improve chemotherapeutic treatment [70,71]. The hypothesis obviously needs further confirmation but may be considered as a potential novel application in personalized treatment of cancer.

## Conclusion

Our model of tumorigenesis, relying on TP53 Arg72Pro, may hold true only in selected populations of patients. In areas of high HCC incidence (Far East, Sub-Saharan Africa), populations are, indeed, exposed to potent risk factors such as perinatal infection with hepatitis B virus or exposure to aflatoxin B1. Such conditions, known to induce major genomic alterations in HCC, are presumably dwarfing the subtle impact of rs1042522 [72]. Cases of primary liver cancers with somatic changes depending primarily on *TP53* R72P genotype might be confined to low incidence areas such as WNA, Middle East, Indian sub-continent or South America. In a research field dominated by clinical and biological studies undertaken on Far-Eastern patients, our work emphasizes the necessary space for a research conducted on alternative populations affected with specific tumor processes.Such studies may provide valuable information for the understanding of a disease which natural history and biology remain amazingly diverse from one world region to another.

## Material and methods

### Tissue specimens and DNA extraction

Sixty-three liver tissues including forty five HCC and seventeen matching corresponding non-tumorous liver tissues were obtained from WNA patients who underwent a liver surgery at University hospitals in Morocco (n = 41) and Tunisia (n = 4). Clinico-pathological features of patients are described in Table 1. In addition, PBMCs of thirty-five patients with chronic viral hepatitis B or C (27 HBV+ and 8 HCV+) but without tumors were collected for comparative methylation analysis with 25 PBMCs samples from patients with HCCs. All samples have been already published in previous surveys [2]. The research was conducted with the informed consent of the patients in accordance to the recommendations issued by the conference of Helsinki. It was approved by the Ethics Committees of the Faculty of Medicine of Casablanca and that of the Tunisian Ministry of Health. DNA was extracted from frozen tissues and blood samples as previously described [73].

### SNP genotyping, Screening of mutations and LOH

Data of SNP in *TP53* (rs1042522) were previously published [74]. Exons 4–10 of *TP53*, exons 2–6 of *CTNNB1,* exons 2–8 of *AXIN1* were analyzed for the presence of point mutations by PCR-sequencing as previously described (2). Six chromosomes (1p, 4q, 6q, 8p, 9p, 17p) considered as “hotspots” of deletions in HCC, were tested for loss of heterozygosity (LOH) according to the protocol previously described [72].

### Sodium bisulfite treatement

PBMCs, non-tumorous and tumorous genomic DNA samples were subjected to bisulfite treatment using Epitect® Bisulfite Kit (Qiagen, Courtabœuf, France) according to manufacturer’s instructions. WI38 cells DNA were used as positive control of methylation after treatment with 12 units SssI methyltransferase (NE Biolabs, Ipswich, MA) in the presence of S-adenosyl-methionine 32 μM.

### Methylation assessment

Methylation status was assessed for 10 loci including eight cancer-related genes and two miRNAs coding loci, methylation presence was determined by methyl-specific PCR (MSP) for *RIZ1*, *RASSF1*, *SOCS1*, *TERT*, *TNFRSF10C* and *GSTP1* genes (Additional file 1: Table S4), and using COBRA for *NRG1*, *CLU*, *mir-203* and *mir-663* genes (Additional file 1: Table S5). Quantification of LINE-1 methylation was performed by COBRA as well according to the method described previously [75]. Variations in bands intensity were analyzed by densitometry using a CCD camera and the GeneTools software (Syngene, Ozyme, Saint-Quentin-en-Yvelines, France). A 50% increase of methylated product in tumorous DNA as compared with non-tumorous was considered as the positive threshold. The methylation index (MethIndex) was defined as the ratio of the number of loci found methylated (*Met(+)Loci*) on the total number of informative loci in a given sample(*[Met(+) + Met(−)]Loci*): *Met(+)-Loci÷[Met(+) + Met(−)]Loci*.

### Methyl-specific PCR (MSP)

Each PCR reaction was performed in a 25 μl final volume containing 100 ng of genomic DNA, 1X PCR buffer, 1.5 mM MgCl_2_, 200 mMdNTP, 20 pmol of each primer, 5 units of AmpliTaq GOLD DNA polymerase (Applied Biosystem, Saint-Aubin, France). Reactions were subjected to 94°C for 5 min, followed by 40 cycles at 95°C for 1 min, 50-60°C for 1 min and 72°C for 1 min and final extension at 72°C for 7 min on a MyCycler thermal cycler (Bio-Rad, Marnes-la-Coquette, France). PCR products were separated on 3% agarose gels stained with ethidium bromide (Euromedex, Souffelweyersheim, France) as shown in Figure 1A [76-78]. All primer sequences are provided in Additional file 1: Table S4. Primers used for *GSTP1*, *RASSF1*, *RIZ1*, and *hTERT* analyses have been previously published [79-81].

### Combined bisulfite restriction assay (COBRA)

PCR reactions were performed as described in MSP method with modification of Taq DNA polymerase using Platinium® Taq DNA Polymerase (Life Technologies, Illkirch, France). A volume of 15 μl of PCR products was then digested with suitable restriction enzymes (Fermentas, Life Technologies, Illkirch, France, Additional file 1: Table S5). PCR products were separated on 2.5% stained agarose gels and results were determined referring to the density of bands related to those of controls (Figure 1B). Primers used for miR-203 and LINE1 analyses have been previously published [12,75].

### Cell cultures, western blot analysis, MTT assay

HCC derived cell lines analyzed have been described previously [82] (Additional file 4). The cells were cultured in DMEM (Life Technologies, Saint-Aubin, France) supplemented with FBS (10%, Biowest, Nuaille, France) and antibiotic-antimycotic. Cells were incubated and maintained at 37°C with humified air and 5% CO_2_ (Binder, Tuttlingen, Germany). Cells at 50-60% confluence were treated for 6-96 h at various concentrations of doxorubicin (Selleck chemicals, Euromedex, Souffelweyersheim, France) and/or decitabine (Fluka, Saint-Quentin Fallavier, France). DNA, RNA, and proteins were extracted from 6-well plates (TPP, Dominique Dutscher, Brumath, France). Cell culture experiments were performed at least three times. For protein analysis, cells were washed in cold phosphate buffered saline (PBS), lysed in Laemmli buffer as described previously and samples loaded on Criterion XT precast gels (Bio-Rad, Marnes-la-Coquette, France) [83]. Western-blots were carried out with a Trans-blot Turbo transfer system as indicated by the manufacturer (Bio-Rad). Blots were probed with primary antibody against p53 (Santa-Cruz, CliniSciences, Nanterre, France sc-6243, 1/1000) and vinculin (Abcam, Cambridge, UK, ab18058, 1/500) or actin (Sigma, Saint-Quentin Fallavier, France, a2066, 1/5000). Cell viability was measured by MTT (3-(4,5-dimethylthiazol-2-yl)-2,5-diphenyl-tetrazolium bromide, Euromedex) assay after seeding in flat bottom 96-well plates (TPP) and treatment for various time length. Formazan crystal resulting from MTT reduction was solubilized in acid isopropanol and absorbance measured at 570 nm on a Fluostar Omega plate reader (BMG Labtech, Champigny/Marne, France).

### Quantitative RT-PCR

Total RNA was obtained from cell culture by direct lysis in Tri-Reagent (Sigma). One microgram of RNA was reverse transcribed using a high-capacity cDNA Archive Kit (Applied Biosystems, Villebon, France) according to the manufacturer’s specifications. Expression of 15 genes involved in DNA methylation (*DNMT3A-B-L*, *DNMT1*) and demethylation (*TET1*-*3*, *APOBEC1*, *APOBEC3A-B-C-H*, *AID*, *SMUG* and *TDG*) processes was analyzed. Two known p53-responsive genes (p21-*CDKN1A*, *GADD45*) were included in the analysis. Real-time PCR was performed in a CFX96 qPCR machine (Bio-Rad). All samples were measured in triplicate. The PCR volume of 10 μl included 20 ng of RT product, 1× TaqMan Universal PCR master mix and 1 μl of pre-validated Taqman Gene Expression Assay (TaqMan FG Shelf, Applied Biosystems, see Additional file 1: Table S6). The reactions were incubated in a 96-well optical plate at 95°C for 10 min, followed by 40 cycles of 95°C for 15 sec and 60° for 1 min. The Ct data was determinate using default threshold settings. The threshold cycle (Ct) is defined as the fractional cycle number at which the fluorescence passes the fixed threshold. For data analysis, gene expression values were determined using the calculation of the relative quantitation (RQ) of target genes normalized to a calibrator corresponding to 5 normal livers. RQ calculation was performed using the DeltaCT method with the geometric mean of three reference genes (*TRIM44*, Hs00214040_m1, *HMBS*; Hs00609297_m1 and *LMF2*; Hs00611068_m1) as reference [84]. The three references genes were selected among 12 constant genes arising from a previous array analysis of 70 HCC samples and 9 normal livers to which were applied algorithms described previously [85].

### Quantitative real-time PCR on genomic DNA

QPCR coupled Methyl Sensitive Restriction Enzyme (MSRE) reactions were carried out as described by Melnikof *et al.* [37]*.* Genomic DNA was extracted as described previously and quantified using NanoDrop ND-1000 (NanoDrop, Wilmington, USA) [82]. All digestions were performed with HpaII, a methylation sensitive restriction enzyme. An amount of 200 ng of genomic DNA was digested 16 h at 37°C in a final volume of 100 μl containing 10 units of HpaII or HhaI. After incubation, each digested sample was diluted 5-fold with sterile water and incubated at 65°C for 15 min to inactivate the enzyme. QPCR was performed in triplicate on a Bio-Rad CFX-96 system (Bio-Rad) with 10 μl of reaction mixture containing 4 ng of digested DNA, 5 μl of 2X Sso Advanced SYBR Green Supermix (Bio-Rad) and 5 pmoles of each primer. Primers at CMYA5 gene, mapping in 5q21.1, a region non-affected by copy number changes in HCC, were used as reference. QPCR conditions included a denaturation step at 98°C for 2 min followed by 40 cycles at 98°C for 5 sec and 60°C for 1 min [86]. Results were analyzed using the CFX Manager and Precision Melt Analysis software (Bio-Rad). Primers are provided on Additional file 1: Table S7.

### Statistical analysis

Instat software (GraphPad Software Inc, La Jolla, CA, USA) was used for statistical analysis of the data with the Chi-square test, Fisher’s exact test, Student’s *t*-test, the Mann–Whitney test, ANOVA test and Kruskal-Wallis test as appropriate. The cut-off value for significance was of P < 0.05. All tests were two-sided.

## Additional files

Additional file 1: Table S1. Functions of the genes investigated for DNA methylations in HCC from Western North-African patients. **Table S2.** Correlation matrix of the methylated loci as detected in North-African Hepatocellular Carcinoma. **Table S3.** Genetic features associated with abnormal DNA methylation in North-African Hepatocellular Carcinoma. **Table S4.** Methylation-specific PCR primers. Genome locations of primer sequences are given according to those provided by the Genome browser gateway. (http://genome-euro.ucsc.edu/cgi-bin/hgGateway?db=hg11&redirect=auto&source=genome.ucsc.edu). Um: amplifies unmethylated DNA, M: amplifies methylated DNA. **Table S5.** COBRA assay primers. Genome locations of primer sequences are given according to those provided by the Genome browser gateway. (http://genome-euro.ucsc.edu/cgi-bin/hgGateway?db=hg11&redirect=auto&source=genome.ucsc.edu). **Table S6.** Taqman Gene Expression Assay (PE Applied Biosystems). **Table S7.** MSRE-qPCR primers. Genome locations of primer sequences are given according to those provided by the Genome browser gateway (http://genome-euro.ucsc.edu/cgi-bin/hgGateway?db=hg11&redirect=auto&source=genome.ucsc.edu). Additional file 2: Figure S1. (A) DNA methylation levels for the loci studied. No difference was statistically significant. (B) Methylation Index at 9 loci for DNA extracted from peripheral lymphocytes in patients with or without primary liver cancer. (C) Non-supervised hierarchical clustering of DNA methylation in PBMCs does not detect any difference between patients with or without liver cancer. Additional file 3: Figure S2. Map of six among the seven promoters analyzed by MSRE-qPCR. Positions of primers are indicated by double arrows. Nucleotide position of primers is defined according to transcription Start site (↱1). Restriction sites are shown above the gene. Exons are symbolized at the scale by rectangles. *BIRC5* assay is not shown. Additional file 4:
**Supplementary data.**

## Abbreviations

ANOVA: Analysis of variance
APOBEC: Apolipoprotein mRNA editing enzyme
Arg: Arginine
CIN: Chromosomal instability
CLU: Clusterin
COBRA: Combined bisulfite restriction assay
CTNNB1: Beta-catenin
DNMT: DNA methyltransferase
GSTP1: Glutathione S-transferase pi 1
HBV: Hepatitis B virus
HCC: Hepatocellular carcinoma
HCV: Hepatitis C virus
LINE-1: Long interspersed nuclear element 1
LOH: Loss of heterozygosity
mir: microRNA
MSP: Methyl-specific PCR
NRG1: Neuregulin1
NTL: Non-tumorous liver
PBMCs: Peripheral blood mononuclear cells
Pro: Proline
SOCS1: Suppressor of cytokine signaling 1
TET: Tet methylcytosinedioxygenase
TP53: Tumor protein 53
TSG: Tumor suppressor gene
R72P: Arginine72Proline
RASSF1: Ras association domain family member 1
RIZ1: Retinoblastoma-interacting zinc-finger protein 1
SNP: Single nucleotide polymorphism
WNA: Western North-Africa
