# Supplementary material for: TP53 R72P polymorphism modulates DNA methylation in hepatocellular carcinoma
Source: Mol Cancer. 2015 Apr 2;14:74. doi: 10.1186/s12943-015-0340-2 (PMC4393630; doi:10.1186/s12943-015-0340-2)
Supplement: Additional file 1: Table S1. — Functions of the genes investigated for DNA methylations in HCC from Western North-African patients. Table S2. Correlation matrix of the methylated loci as detected in North-African Hepatocellular Carcinoma. Table S3. Genetic features associated with abnormal DNA methylation in North-African Hepatocellular Carcinoma. Table S4. Methylation-specific PCR primers. Genome locations of primer sequences are given according to those provided by the Genome browser gateway. (http://genome-euro.ucsc.edu/cgi-bin/hgGateway?db=hg11&redirect=auto&source=genome.ucsc.edu). Um: amplifies unmethylated DNA, M: amplifies methylated DNA. Table S5. COBRA assay primers. Genome locations of primer sequences are given according to those provided by the Genome browser gateway. (http://genome-euro.ucsc.edu/cgi-bin/hgGateway?db=hg11&redirect=auto&source=genome.ucsc.edu). Table S6. Taqman Gene Expression Assay (PE Applied Biosystems). Table S7. MSRE-qPCR primers. Genome locations of primer sequences are given according to those provided by the Genome browser gateway (http://genome-euro.ucsc.edu/cgi-bin/hgGateway?db=hg11&redirect=auto&source=genome.ucsc.edu). [file 12943_2015_340_MOESM1_ESM.docx]

| **Gene Symbol** | **Full name** | **Aliases** | **MIM** | **Cytoband** | **Function/pathway** |
| --- | --- | --- | --- | --- | --- |
| *CLU* | Clusterin | CLI; AAG4; APOJ; CLU1; CLU2; KUB1; SGP2; APO-J; SGP-2; SP-40; TRPM2; TRPM-2; NA1/NA2 | 185430 | 8p21-p12 | Chaperone, ERK1/2 signaling modulator |
| *GSTP1* | Glutathione S-transferase pi 1 | PI; DFN7; GST3; GSTP; FAEES3; HEL-S-22 | 134660 | 11q13 | Glutathione metabolism, Detoxication |
| *MIR203A* | microRNA 203a | MIR203; MIRN203; miR-203; miRNA203; hsa-mir-203a | 611899 | 14q32.33 | Oncosuppressive, Wnt signaling inhibitor |
| *MIR663A* | microRNA 663a | MIR663; MIRN663; hsa-mir-663; hsa-mir-663a | 724033 | 20p11.1 | Direct or indirect regulation of TGFB1, Bax, Fas |
| *NRG1* | Neuregulin 1 | GGF; HGL; HRG; NDF; ARIA; GGF2; HRG1; HRGA; SMDF; MST131; MSTP131; NRG1-IT2 | 142445 | 8p12 | Embryonic development, resistance to tyrosine-kinase inhibitors |
| *RASSF1A* | Ras association domain family member 1 | 123F2; RDA32; NORE2A; RASSF1; REH3P21 | 605082 | 3p21.3 | Inhibits cyclin D1 accumulation |
| *RIZ1* | PR domain containing 2, with ZNF domain | PRDM2; KMT8; RIZ1; RIZ2; MTB-ZF; HUMHOXY1 | 601196 | 1p36 | Histone methyltransferase, binds to RB1, heme-oxygenase and estrogen receptor |
| *SOCS 1* | Suppressor of cytokine signaling 1 | JAB; CIS1; SSI1; TIP3; CISH1; SSI-1; SOCS-1 | 603597 | 16p13.13 | Negative regulation of JAK-STAT cascade |
| *TERT* | Telomerase reverse transcriptase | TP2; TRT; CMM9; EST2; TCS1; hTRT; DKCA2; DKCB4; hEST2; PFBMFT1 | 187270 | 5p15.33 | Telomere maintenance, oncogenic |
| *TNFRSF10C* | Tumor necrosis factor receptor superfamily, member 10c | LIT; DCR1; TRID; CD263; TRAILR3; TRAIL-R3; DCR1-TNFR | 603613 | 8p22 | Programmed cell death |

Table S1: Functions of the genes investigated for DNA methylation in HCC from Western North-African patients

|  | **RASSF1** | **SOCS1** | **miR203** | **hTERT** | **TNFRSF10C** | **NRG1** | **GSTP1** | **LINE** | **CLU** | **Meth Index** |
| --- | --- | --- | --- | --- | --- | --- | --- | --- | --- | --- |
| **RIZ1** | 0.00519 | 0.00167 | 0.00344 | 0.0244 | *0.06421* | 0.0133 | *0.07449* | 0.00844 | ns | <0.0001 |
| **RASSF1** |  | 0.01068 | 0.00496 | 0.0358 | *0.07737* | ns | 0.04966 | ns | ns | <0.0001 |
| **SOCS1** |  |  | 0.04454 | *0.0913* | ns | ns | ns | ns | ns | <0.0001 |
| **miR203** |  |  |  | ns | ns | ns | ns | 0.02291 | ns | 0.0031 |
| **hTERT** |  |  |  |  | ns | *0.0526* | ns | ns | ns | 0.0004 |
| **TNFRSF10C** |  |  |  |  |  | 0.01179 | ns | ns | ns | <0.0001 |
| **NRG1** |  |  |  |  |  |  | ns | ns | ns | 0.0003 |
| **GSTP1** |  |  |  |  |  |  |  | ns | ns | 0.0105 |
| **LINE** |  |  |  |  |  |  |  |  | ns | ns |
| **CLU** |  |  |  |  |  |  |  |  |  | ns |

Table S2: Correlation matrix of the methylated loci as detected in North-African Hepatocellular Carcinoma.

| ***Clinical and Genetic features*** | ***Locus*** | ***Proportions*** | ***RR*** | ***(95%CI)*** | ***P value*** |
| --- | --- | --- | --- | --- | --- |
|  |  | ***Methylated vs Unmethylated*** |  |  |  |
| **Genetic polymorphisms** |  |  |  |  |  |
| TP53 codon 72 PROPRO | *SOCS1* | 3 vs 29 % | 0.117 | **0.014 to 0.92** | **0.020** |
| **Point mutations** |  |  |  |  |  |
| mutation presence | *RIZ1* | 53 vs 16 % | 5.9063 | **1.41 to 24.72** | **0.0120** |
| **Chromosome Instability** |  |  |  |  |  |
| LOH4q | *miR-203* | 54 vs 22 % | 4.2857 | **1.0023 to 18.32** | **0.0425** |
| LOH17p | *RASSF1* | 44 vs 12 % | 5.5 | **1.02 to 29.47** | **0.0303** |
|  | *RIZ1* | 52 vs 13 % | 7.125 | **1.51 to 33.43** | **0.0095** |

Table S3: Genetic features associated with abnormal DNA methylation in North-African Hepatocellular Carcinoma.

| ***Gene*** |  | ***Primer sequences*** | ***Corresponding native sequence on GRCh37/hg19*** | ***Genomic location*** | ***Size*** | ***Ann T°*** |
| --- | --- | --- | --- | --- | --- | --- |
| *RASSF1A* | RAU | F: TTTGGTTGGAGTGTGTTAATGTG | F:CCCGGCTGGAGCGTGCCAACGCG | chr3:50378059-50378166 | 108pb | 55°C |
|  |  | R: CAAACCCCACAAACTAAAAACAA | R: CGGGCCCCGCGGGCTGGAAGCGG |  |  |  |
|  | RAM | F:GTGTTAACGCGTTGCGTATC | F:GCGTGCCAACGCGCTGCGCATC | chr3:50378061-50378156 | 96pb | 48°C |
|  |  | R:AACCCCGCGAACTAAAAACGA | R: GGCCCCGCGGGCTGGAAGCGG |  |  |  |
| *GSTP1* | GSTP1 Um | F: GATGTTTGGGGTGTAGTGGTTGTT | F:GACGCCCGGGGTGCAGCGGCCGCC | chr11:67351139+67351235 | 97pb | 52°C |
|  |  | R: CCACCCCAATACTAAATCACAACA | R: CCGCCCCAGTGCTGAGTCACGGCG |  |  |  |
|  | GSTP1 M | F: TTCGGGGTGTAGCGGTCGTC | F:CCCGGGGTGCAGCGGCCGCC | chr11:67351143+67351233 | 91pb | 52°C |
|  |  | R: GCCCCAATACTAAATCACGACG | R: GCCCCAGTGCTGAGTCACGGCG |  |  |  |
| *RIZ1* | RP291U | F:TGGTGGTTATTGGGTGATGGT | F:TGGTGGCCATTGGGCGACGGC | chr1:14026570+14026744 | 176 pb | 52°C |
|  |  | R:ACTATTTCACCAACCCCAAGA | R: GCTGTTTCGCCGGCCCCGGCG |  |  |  |
|  | RP291M | F:GTGGTGGTTATTGGGCGACGGC | F:GTGGTGGCCATTGGGCGACGGC | chr1:14026569+14026744 | 175pb | 52°C |
|  |  | R:GCTATTTCGCCGACCCCGACG | R: GCTGTTTCGCCGGCCCCGGCG |  |  |  |
| *TNFRSF10C* | Um | F:TTTTTTTATGTGTATGAATTTAGTTAATG | F:CCTCTCCACGCGCACGAACTCAGCCAACG | chr8:22960453+22960587 | 135pb | 55°C |
|  |  | R:AACCATCAAACAACCAAAACAC | R: GGCCATCAGGCGGCCGGGGCGC |  |  |  |
|  | M | F:TTACGCGTACGAATTTAGTTAAC | F:CCACGCGCACGAACTCAGCCAAC | chr8:22960458+22960584 | 127pb | 59°C |
|  |  | R:CATCAAACGACCGAAACG | R: CATCAGGCGGCCGGGGCG |  |  |  |
| *hTERT* | Um | F:AGTTTTGGTTTTGGTTATTTTTGT | F:AGCCCTGGCCCCGGCCACCCCCGCG | chr5:1295025-1295129 | 105pb | 56°C |
|  |  | R:AACGTAACCAACGACAACACCT | R: AACGTGGCCAGCGGCAGCACCT |  |  |  |
|  | M | F:AGTTTTGGTTTCGGTTATTTTCGC | F:AGCCCTGGCCCCGGCCACCCCCGCG | chr5:1295025-1295129 | 105pb | 62°C |
|  |  | R:AACGTAACCAACGACAACACCT | R: AACGTGGCCAGCGGCAGCACCT |  |  |  |
| *SOCS 1* | Um | F:GGTTTTGGGATTTATGAGTATTTGTG | F:GGCCTCGGGACCCACGAGCATCCGCG | chr16:11348776-11348979 | 204pb | 58SD |
|  |  | R:CCCACAATAACCACAATACACTAACA | R: CCCACGGTGGCCACGATGCGCTGGCG |  |  |  |
|  | M | F:TTCGCGTGTATTTTTAGGTCGGTC | F:TCCGCGTGCACTTTCAGGCCGGCC | chr16:11348800-11348959 | 160pb | 64°C |
|  |  | R:CGACACAACTCCTACAACGACCG | R: CGGCACAGCTCCTGCAGCGGCCG |  |  |  |

Table S4: Methylation-specific PCR primers. Genome locations of primer sequences are given according to those provided by the

Genome browser gateway. (<http://genome-euro.ucsc.edu/cgi-bin/hgGateway?db=hg11&redirect=auto&source=genome.ucsc.edu>).

Um: amplifies unmethylated DNA, M: amplifies methylated DNA.

| ***Gene*** | ***Primer sequence*** | ***Corresponding native sequence on GRCh37/hg19*** | ***Genomic location*** | ***Amplimere Size*** | ***Ann T°*** | ***Restriction Enzyme*** |
| --- | --- | --- | --- | --- | --- | --- |
| *NRG1* | F:TTTGATTTTGTTTGTAGTGAT | F:TCTGATCCTGTTTGCAGTGAT | chr8:32504627+32504890 | 261pb | 52°C | **BstUI** |
|  | R:AAACTAAAAAACATATTTCTC | R:AAACTGAAAGGCATGTTTCTC |  |  |  |  |
| *CLU* | F:GGTTTTGTTTTTTTGGTATGATAAAG | F:GGCTTTGTCTCTCTGGCATGACAAAG | chr8:27469149-27469447 | 299pb | 60°C | **HpHI** |
|  | R:AAAATTCCCCTTCCTAAAATAATTC | R:GGGGTTCCCCTTCCTGAAATGGTTC |  |  |  |  |
| *mir203* | F:TTGGGTTTAGTGGTTTTTAATAGTTT | F:CTGGGTCCAGTGGTTCTTAACAGTTC | chr14:104583760+104584086 | 327pb | 55°C | **BstUI** |
|  | R:AACRATTCCCACAACACA | R:AGCGGTTCCCACAGCACA |  |  |  |  |
| *mir663* | F:TTTTTTYGAGTAAGGGGAGGATTT | F:CTTTCCGAGTAAGGGGAGGATCC | chr20:26188989-26189299 | 310pb | 55°C | **RsaI** |
|  | R:AAACTCAACRCCAAAACCACAT | R:AAACTCAACGCCAGGGCCACAT |  |  |  |  |
| *Line1* | F:TTGAGTTGTGGTGGGTTTTATTTAG |  | multiple | 413pb | 50°C | **Cac8I** |
|  | R:TCATCTCACTAAAAAATACCAAACA |  |  |  |  |  |

Table S5: COBRA assay primers. Genome locations of primer sequences are given according to those provided

by the Genome browser gateway.

(<http://genome-euro.ucsc.edu/cgi-bin/hgGateway?db=hg11&redirect=auto&source=genome.ucsc.edu>).

| ***Gene Symbol*** | **Reference** |
| --- | --- |
| *DNMT1* | Hs00154749_m1 |
| *DNMT3A* | Hs01027166_m1 |
| *DNMT3B* | Hs01003405_m1 |
| *DNMT3L* | Hs00203536_m1 |
| *APOBEC3A* | Hs00377444_m1 |
| *APOBEC3B* | Hs00377444_m1 |
| *APOBEC3C* | Hs00828074_m1 |
| *APOBEC3H* | Hs00419665_m1 |
| *TET1* | Hs00286756_m1 |
| *TET2* | Hs00325999_m1 |
| *TET3* | Hs00379125_m1 |
| *AID* | Hs00757808_m1 |
| *TDG* | Hs00702322_s1 |
| *SMUG1* | Hs00204820_m1 |
| *APOBEC1* | Hs00242340_m1 |
| *TP53* | Hs01034249_m1 |
| *CDKN1A* | Hs00355782_m1 |
| *GADD45A* | Hs00169255_m1 |
|  |  |

Table S6 : Taqman Gene Expression Assay (PE Applied Biosystems)

| ***Primer*** | ***sequence*** | ***size*** | ***Genomic location*** | ***HpaII sites*** |
| --- | --- | --- | --- | --- |
| *cdc25f* | ACCATTTCCAGAGCAAGCAC | 182bp | chr5:137667479-137667660 | 0 |
| *cdc25r* | CCTCTGAGCAAGAATATCAACAGCC |  |  |  |
| *BIRC5f* | CGTTCTTTGAAAGCAGTCGAG | 190bp | chr17:76210132+76210321 | 1 |
| *BIRC5r* | GAGCGCACGCCCTCTTAG |  |  |  |
| *MGMTf* | CCGGATATGCTGGGACAG | 96bp | chr10:131265474+131265569 | 1 |
| *MGMTr* | GACACTCACCAAGTCGCAAA |  |  |  |
| *GSTP1f* | GGGACCCTCCAGAAGAGC | 134bp | chr11:67351188+67351321 | 1 |
| *GSTP1r* | ACTCACTGGTGGCGAAGACT |  |  |  |
| *NRG1f* | CCCGAAACTTGTTGGAACTC | 97bp | chr8:32405825+32405921 | 2 |
| *NRG1r* | AGGTTATCACCGTCCTGCTC |  |  |  |
| *RASSF1f* | AGCCTGAGCTCATTGAGCTG | 130bp | chr3:50378097-50378226 | 1 |
| *RASSF1r* | ACCAGCTGCCGTGTGG |  |  |  |
| *SOCS1f* | ACTTCCGCACATTCCGTTC | 186bp | chr16:11348990-11349175 | 1 |
| *SOCS1r* | TAAGGGCGAAAAAGCAGTTC |  |  |  |
| *CLUf* | CCTGGCAGAGAGGTGTTTCT | 366bp | chr8:27469106-27469471 | 1 |
| *CLUr* | ATCTGAGCTCACCCATTTGC |  |  |  |
| *CMYA5f* | CCACACACAACCGAGATGAC | 102bp | chr5:79027160+79027261 | 0 (negative control) |
| *CMYA5r* | TTCCAACTCGACTGCCTCTT |  |  |  |

Table S7 : MSRE-qPCR primers. Genome locations of primer sequences are given according to those provided by the Genome browser gateway (http://genome-euro.ucsc.edu/cgi-bin/hgGateway?db=hg11&redirect=auto&source=genome.ucsc.edu).
