# Supplementary figures and images for: TP53 R72P polymorphism modulates DNA methylation in hepatocellular carcinoma
Source: Mol Cancer. 2015 Apr 2;14:74. doi: 10.1186/s12943-015-0340-2 (PMC4393630; doi:10.1186/s12943-015-0340-2)

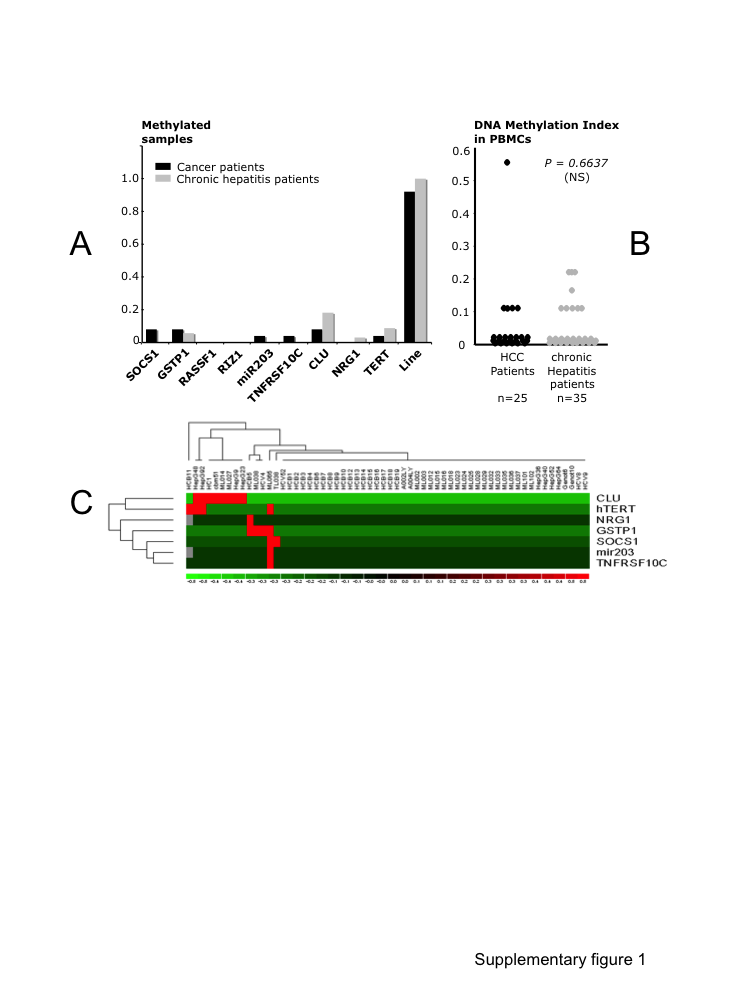

Supplement: Additional file 2: Figure S1. — (A) DNA methylation levels for the loci studied. No difference was statistically significant. (B) Methylation Index at 9 loci for DNA extracted from peripheral lymphocytes in patients with or without primary liver cancer. (C) Non-supervised hierarchical clustering of DNA methylation in PBMCs does not detect any difference between patients with or without liver cancer. [file 12943_2015_340_MOESM2_ESM.tiff]

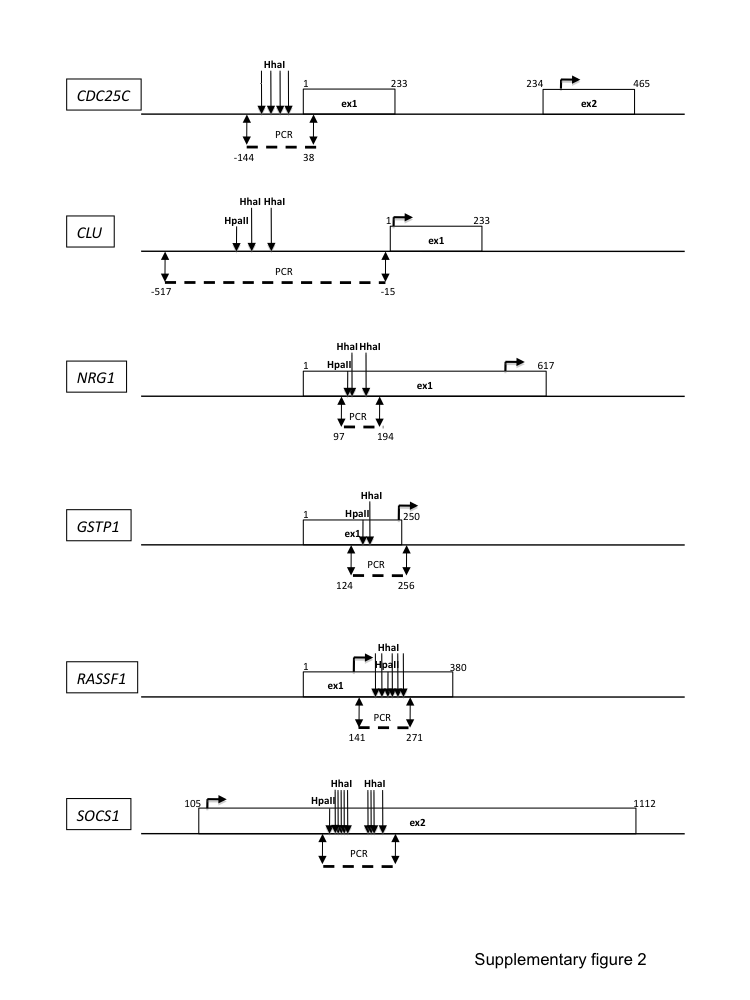

Supplement: Additional file 3: Figure S2. — Map of six among the seven promoters analyzed by MSRE-qPCR. Positions of primers are indicated by double arrows. Nucleotide position of primers is defined according to transcription Start site (↱1). Restriction sites are shown above the gene. Exons are symbolized at the scale by rectangles. BIRC5 assay is not shown. [file 12943_2015_340_MOESM3_ESM.tiff]
