# Supplementary material for: TP53 R72P polymorphism modulates DNA methylation in hepatocellular carcinoma
Source: Mol Cancer. 2015 Apr 2;14:74. doi: 10.1186/s12943-015-0340-2 (PMC4393630; doi:10.1186/s12943-015-0340-2)
Supplement: Additional file 4: — Supplementary data. [file 12943_2015_340_MOESM4_ESM.doc]

Supplementary Data

TP53 R72P (rs1042522) modulates DNA methylation

in Hepatocellular Carcinoma

Khadija Rebbani^1,2^, Agnès Marchio^1^, Sayeh Ezzikouri^2^, Rajaa Afifi^3^, Mostafa Kandil^4^, Olfa Bahri^5^, Henda Triki^5^, Abdellah Essaid El Feydi^3^, Anne Dejean^1^, Soumaya Benjelloun^2^, Pascal Pineau^1*^

^1^Unité d’Organisation Nucléaire et Oncogenèse, INSERM U993, Institut Pasteur, Paris, France,

^2^Laboratoire des Hépatites Virales, Institut Pasteur du Maroc, Casablanca, Morocco.

^3^Service de Médecine C-Gastroentérologie, CHU Ibn-Sina, Rabat, Morocco,

^4^Equipe d’Anthropogénétique et de Biotechnologies, Faculté des Sciences Chouaib Doukkali, El Jadida, Morocco.

^5^Laboratoire de Virologie Clinique, Institut Pasteur de Tunis, Tunis, Tunisie

- corresponding author: Unité “Organisation nucléaire etoncogenèse”, Institut Pasteur, 28, rue du Docteur Roux, F-75724 Paris cedex 15, Phone, 33 1 45 68 88 24, fax, 33 1 45 68 89 43, email: [**pascal.pineau@pasteur.fr**](mailto:pascal.pineau@pasteur.fr)

Material and Methods

Cell lines

Two cell lines (Huh6 and Mahlavu) were obtained from Marie-Louise Michel in the 1980’s decade and five others collected from various places in 1998-99. They have been characterized for homozygous deletion presence at 238 loci and published previously (1). Briefly, FOCUS cells were obtained from Jake Wands at the Massachussetts General Hospital. SK-Hep1 cells were obtained from Dr Chung-Ming Chang from the Veterans General Hospital in Taipei. Tong-HCC was provided by Dr Pei-Jer Chen at the National Taiwan university Hospital. SNU449 was bought at the Laboratory of Cell Biology, Cancer Research Institute, Seoul National University College of Medicine. NuK1 cells were provided by Dr Shuichi Seki from the Third Department of Internal Medicine, Osaka City University Medical School. Finally, Hepa-RG cell line was obtained from Dr Christian Trépo from the INSERM U271 in Lyon in 2010. Sequencing of TP53 on exons 4 to 11 was performed as previously described (2).

References

1. Pineau, P.*, et al.* (2003) Homozygous deletions scanning In tumor cell lines detects previously unsuspected loci. *Int J Cancer*, 106, 216-223.

2. Pineau, P.*, et al.* (2008) In Human Hepatocellular Carcinoma, Chromosome Instability depends on p53 Status and Aflatoxin Exposure. *Mutat Res*, 653, 6-13.
